# Evaluation of Aberdeen Angus Breeding Bulls in the Northern Region of the Republic of Kazakhstan

**DOI:** 10.3390/ani14060894

**Published:** 2024-03-14

**Authors:** Pavel Shevchenko, Jan Miciński, Inna Brel-Kisseleva

**Affiliations:** 1 Department of Food Security and Biotechnology, NLC «Akhmet Baitursynuly Kostanay Regional University», Kostanay 110000, Kazakhstan; pavel87011339688@gmail.com; 2 Department of Sheep and Goat Breeding, Faculty of Animal Bioengineering, University of Warmia and Mazury in Olsztyn, 10-719 Olsztyn, Poland; micinski@uwm.edu.pl

**Keywords:** beef cattle, Aberdeen Angus, proven bull, selection, breeding and genetic parameters

## Abstract

**Simple Summary:**

The Republic of Kazakhstan showcases a diverse range of specialized breeds of beef cattle, both local and exotic. Among these breeds, Aberdeen Angus exhibits the most promise for producing superior quality beef. This is due to its harmonious exterior and constitutional features, precocity, and endurance, which render it highly adaptable to the sharply continental climate of the northern region of Kazakhstan. Given the current surge in demand for high-quality marbled beef derived from Aberdeen Angus cattle, it would be judicious to undertake large-scale breeding of this breed in enterprises of varying ownership structures while utilizing elite breeding bulls for reproduction. In this study, a scientifically rigorous approach was adopted to select the best breeding bulls by assessing the quality of their offspring, daughters, and cows in a comparative context, which underscores the efficacy of using top-quality animals of imported breeding. The results of this research can be utilized in the development of industrial-scale plans to enhance local beef cattle, as well as in private farms with Aberdeen Angus cattle, with a view to creating new genotypes, highly productive lines, and herds in the Republic of Kazakhstan.

**Abstract:**

In this study, we evaluated the progeny quality of proven bulls of the Aberdeen Angus breed in different selections. Reliable information about the productive qualities of the daughters and cows of proven bulls is limited in Kazakhstan’s scientific literature. We aimed to identify bull-improvers by comparatively evaluating cows of different genotypes in terms of productive qualities. The study was conducted in the period of 2021–2023, during which four groups of cows were formed based on origin. In conclusion, the high influence of inheritance of breeding features from bull-improvers should be highlighted: 78.6% to the daughters of cows of group I (Estonian selection from Kolos-firma LLP: EE 14465116 ABAJA PORTOS 95283, DK 1989501341 FREDERIK 95220, and EE 16966079 ABAJA PATRICK 95305) and 74.5% to the daughters of cows of group III (Canadian selection from Vishnevskoe LLP: BH BRUIN 54X 1644270 and JL DISTRICT 0311 1594050). There was less influence from proven bulls of deteriorators of the Kazakh selection: 21.4% to the daughters of cows of group II from Kolos-firma LLP (KZP156547191) and 25.5% to the daughters of cows of group IV from Vishnevskoe LLP (Henri KZT157789649 and Argus KZT155778964). Therefore, it is recommended that valuable genotypes of imported selection are used in breeding work in the Kostanay and North Kazakhstan regions for the improvement of Aberdeen Angus cattle.

## 1. Introduction

The Republic of Kazakhstan places significant emphasis on ensuring food security, with particular attention given to increasing domestic livestock production. Of all the livestock products, beef is the most valuable and irreplaceable food product, and beef cattle breeding plays a crucial role in the production of this commodity [1,2,3]. Modern directions of agricultural production development imply the further improvement of existing and the creation of new highly productive breeds, types, and lines of beef cattle, which contribute to increasing the efficiency of the industry and obtaining high-quality products [4,5,6]. Due to their economic and biological features, meat cattle breeds are of great value and have high potential for increasing meat production [7,8,9,10,11].

Breeding cattle for meat involves various factors that can impact their hereditary and non-hereditary traits, including changes in physique and breeding and productive qualities. As a result, continuous study, analysis, and monitoring of these traits are necessary to ensure the best outcomes in breeding and production.

For instance, the evaluation of breeding bulls is especially critical as it can significantly impact the speed of the reproductive process and the creation of highly productive animals that are well suited to innovative technologies based on large-scale breeding [12,13,14]. Additionally, the selection of cows and heifers in respect of the uterine composition is crucial in achieving breeding herds with appropriate productivity, exterior and constitutional features, milk-producing ability, reproductive ability, good maternal qualities, health, and uniformity. All of these factors rely on the provenance of the cattle; according to those in the field, they must be from outstanding proven bulls [15,16,17].

This approach holds promising prospects for creating highly productive types of beef cattle that can thrive in diverse climatic and economic zones within the Republic of Kazakhstan, including the zone of northern Kazakhstan, which is characterized by a sharply continental climate.

In the context of Kazakhstan’s livestock industry, a two-stage assessment of breeding bulls based on the quality of their offspring is employed, as per the instructions for bonitization [18]. It is evident that the current evaluation of breeding bulls based on the quality of their offspring is limited to points awarded for the bonus class. For example, the sire’s class is categorized as follows: “elite record”, 5 points and “elite”, 4 points. To improve Kazakh breeding, conducting a comprehensive assessment of the breeding bulls based on the quality of their offspring through daughters of cows or repair heifers is necessary to identify the best genotypes.

In order to tackle this issue, several other countries, such as the USA and Europe, have adopted various methods to evaluate breeding bulls based on both the quality of their offspring and the level of development of productive qualities that are relevant to meat breeds [19,20,21,22]. By accurately assessing bull performance based on the personal productive characteristics of their offspring, these countries can implement parallel directional selection of cows for breeding, resulting in the objective identification of the best genotypes. Previous studies have demonstrated the effectiveness of such an assessment system in achieving this goal [23,24,25,26].

The development of a scientifically grounded methodology for the evaluation of breeding bulls is crucial in addressing this task. Such an approach would facilitate the formation of a resilient branch of specialized beef cattle breeding in Kazakhstan, which involves the selective breeding of both local and effective imported cattle breeds. This would result in significant productive and economic advantages. Therefore, the implementation of such a methodology is imperative for the advancement of Kazakhstan’s beef cattle breeding industry.

The improvement of the breeding process aimed at enhancing the productive and technological qualities of Aberdeen Angus cattle is a pressing task. The outcomes of this effort can significantly influence the successful development of the agro-industrial complex, especially the meat livestock industry of the Republic of Kazakhstan. Additionally, it offers opportunities for the sustainable domestic breeding of Aberdeen Angus cattle. As such, this knowledge is of utmost importance to the advancement of Kazakhstan’s livestock industry.

The Aberdeen Angus breed has well-established populations of domestic breeding that have gained widespread adoption. To identify the direction of further development of the breed in the Republic of Kazakhstan, analyzing the genealogical composition of proven bulls used in farms is crucial. The breeding stock of such lines possess valuable productive characteristics and play a fundamental role in the development of the breed.

The aim of this research was to study the influence of breeding qualities of proven Aberdeen Angus breed bulls of local selection (Kazakhstan) and imported selection (Canada and Estonia) on the productivity of daughters and mother cows in comparison and to determine the improved proven bull producers using the selection–genetic parameters of the main economically useful traits and the evaluation of phenotypic changes.

## 2. Materials and Methods

### 2.1. Study Area

The northern region of Kazakhstan comprises two large regions, i.e., Kostanay and North Kazakhstan, each containing several districts with numerous settlements, including large livestock enterprises specializing in breeding Aberdeen Angus beef cattle. This breed was selected because it is the primary pedigree breed among priority imported breeds of cattle, such as Hereford, Santa Gertrude, and Obrok, which are used in the production of high-quality beef in the northern region of Kazakhstan.

The study area is located in a region with highly favorable conditions for agriculture, particularly in beef cattle breeding. The climate of the region is characterized by pronounced continental conditions, with hot and dry summers and cold winters with minimal snowfall. During winter, the minimum air temperature often drops to −35–−40 °C, and in some instances, to −45–−50 °C. In summer, the absolute temperature ranges from +41.0 to 45.0 °C. The warm period with an average daily temperature above 0 °C lasts for 195–200 days, from 7–12 April to 19–28 October. These conditions enable the production of high-quality beef.

### 2.2. Research Conditions

The focus of this research was Aberdeen Angus breeding stock, consisting of diverse genotypes from livestock enterprises situated in the northern Kazakhstan region. Specifically, Kolos-firma LLP, located in Kostanay, Kazakhstan, and Vishnevskoye LLP, situated in North Kazakhstan, were chosen for this study, due to the favorable natural and production–economic conditions that they offer for breeding this particular breed of cattle.

In the course of a meticulous selection process, all cows aged 5 years were chosen to be included in the experimental groups, with the majority of these cows having sires which were proven bulls, accounting for more than 10% of the total livestock in two of the analyzed herds, depending on different genotypes. Accordingly, based on the research scheme presented in Table 1, four groups of cows were formed from Kolos-firma LLP and Vishnevskoe LLP. Group I comprised cows belonging to various paternal lines, with proven bulls of Estonian breeding: DK 1989501341 FREDERIK 95220 (n = 11 cows), EBA Eagle Bando 1114 KZP156547172 (n = 17 cows), EE 14465116 ABAJA PORTOS 95283 (n = 10 cows), and EE 16966079 ABAJA PATRICK 95305 (n = 10 bulls). Group II comprised the genotype of the Kazakh breeding bull: KZP156547191 (n = 17 cows). For Vishnevskoe LLP, Group III cows comprised the genotypes of Canadian breeding bulls: BH BRUIN 54X 1644270 (n = 10 cows), S7R BARRISTER 45X 1639080 (n = 13 cows), and JL DISTRICT 0311 1594050 (n = 13 cows). Group IV cows comprised the genotypes of Kazakh breeding bulls: Henri KZT157789649 (n = 10 cows) and Argus KZT155778964 (n = 10 cows).

In the studied farms “Kolos-firm” and “Vishnevskoe”, artificial insemination is used. Conditions of organization of cow calving are seasonal (March–April).

In the research carried out, a variety of sources of information were utilized, including breeding animal cards, summary bonus lists, and in-house research.

We focused on several aspects, including the current size of the breed; the breed’s genealogical structure in the context of the lines present in the studied enterprises; and an analysis of the main productive characteristics of cows, including their live weight, exterior and constitutional features, and milk production dynamics.

The methodology employed adhered to the widely accepted methodological recommendations that have been sanctioned by the Ministry of Agriculture of the Republic of Kazakhstan. These recommendations encompass instructions on the bonitization of beef breeds of cattle from 2014 in the Order of the Ministry of Agriculture of the Republic of Kazakhstan dated 23 October 2014 No. 9818 [27].

When evaluating the indicators “exterior” and “live weight” of cows in the Republic of Kazakhstan, we used the “Scale of evaluation of cows on the complex of features” according to the instruction on the bonitization (evaluation) of beef breeds of cattle according to the “Scale of evaluation of cows on the complex of features” [27].

The evaluation of the indicator “milk-producing ability” of the studied cows was carried out by the indicator “body weight” of their offspring at 6 months of age. This is because the main method of growing young animals after birth involves keeping the calf together with the cow, according to the instruction for the evaluation of beef breeds of cattle [27].

The research work conducted drew upon several sources of information, including breeding animal records, summary bonus lists, and proprietary research data. We examined various aspects, including the current size of the breed, the genealogical structure of the breed in the context of the lines present in the studied enterprises, and an analysis of the main productive characteristics of cows, specifically their live weight, exterior and constitutional features, and milk-producing ability dynamics.

The experimental groups of cows were under the same conditions of feeding, keeping, and care. The farms practiced loose keeping of cows with provision of daily exercise. Provision of cows with fodder corresponded to the normative parameters of adult beef cattle breeding stock per 100 kg of live weight required: when feeding in the winter stall period indoors, 2.3–2.8 kg of dry matter and 1.9–2.4 EFUs (energy feed units) and when feeding in the paddock–fodder yard, 2.6–3.0 kg of dry matter and 2.1–2.6 kg, per 1 EFU, 82–86 g of digestible protein is required [28].

The research and data analysis phases involved the use of several techniques, including monographic analysis, comparative analysis, and theoretical generalization of the obtained results. The primary digital research material was subjected to biometric analysis through the application of variation statistics, which entailed the computation of selection and genetic indicators, such as the correlation coefficient, heritability coefficient, selection differential, and selection effect. These calculations were performed using Microsoft Excel 2019.

1. Calculation of the weighted average was performed using the following formula:

$$X=\frac{\sum Xn}{\sum n}$$

where *X* is the weighted average; ∑*Xn* is the sum of values; ∑*n* is the sum of the number of bulls.

2. We calculated the mean square deviation using the following formula:

$$\sigma =\pm K\sqrt{\frac{\sum fa^{2}}{n}-\left(\frac{\sum f\alpha }{n}\right)}2$$

where *K* is the size of the class gap; *f* is the frequency; *a* is the deviation from the conditional average class; *n* is the number of sampling options.

3. The formula used to determine the confidence score of the weighted average was as follows:

$$Mx=\pm \frac{\sigma }{\sqrt{n}}$$

4. The heritability coefficient in a one-factor dispersion complex was determined using the following formula:

$$h^{2\;}=\frac{Cx}{Cy}$$

where *h*^2^ is the heritability coefficient; *Cx* refers to the indicator of general genotypic diversity; *Cy* refers to the indicator of general phenotypic diversity.

5. The correlation coefficient was calculated using the following formula:

$$r=\frac{\sum fAx\;Ay-nBxBy}{nSxSy}$$

In the given formula, *A_X_* represents the deviation of classes from the conditional middle class based on the first attribute, while *A_y_* represents the same for the second attribute; *f* represents the frequencies in the correlation lattice; *n* represents the number of animals; *B* and *S* were calculated for the series of the first and second signs using the following formulas:

$$B=\frac{\sum fA}{n}S=\sqrt{\frac{\sum fa^{2}}{n}-B^{2}}$$

6. The calculation of the selection differential (*Sd*) was carried out using the following formula:

*Sd* = *X_i_* − *X*

where *Sd* is the selection differential; *X_i_* is the average value of the trait of the test descendants; *X* is the average value of the traits of animals throughout the herd.

7. The calculation of the selection effect (*SE*) was carried out according to the following formula:

$$SE=Sd\cdot \;h^{2}$$

8. The reliability of the difference in average values (*P*) was determined using Student’s table (*td*), and the following formula was used:

$$td=\left(X_{1}-X_{2}\right)/\sqrt{m1^{2}+m2^{2}}$$

where *X*_1_ − *X*_2_ is the difference between the two averages; $\sqrt{m1^{2}+m2^{2}}$ is the difference in the average error.

## 3. Results

The Aberdeen Angus breed of specialized beef cattle has roots from various imported breeding, making it widespread throughout Kazakhstan, particularly in the northern region, which comprises Kostanay and North Kazakhstan [29,30,31]. Populations of Estonian, Canadian, American, Australian, and Kazakh domestic breeding have been established in the Aberdeen Angus breed. The genealogical composition of proven bulls from Estonian, Canadian, and Kazakh breeding was analyzed. An evaluation of proven bulls based on the quality of their offspring through the productivity of daughter cows, with the calculation of breeding and genetic parameters, was conducted. The best genotypes and directions for their further use in the improvement of Kazakh breeding were identified.

Proven bulls are instrumental in improving the herd because they exhibit a higher breeding effect compared to other bulls with the same linear affiliation.

### 3.1. The Current State of Beef Cattle Breeding in Kazakhstan

**Figure 1:**
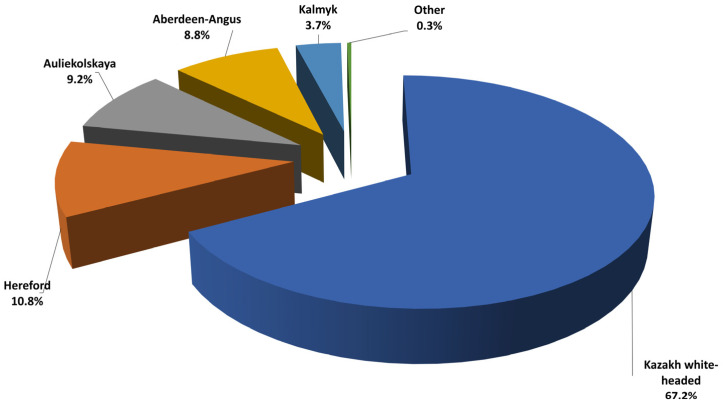
Structure of beef cattle breeds in the Republic of Kazakhstan, %.

The Kazakh market of meat raw materials is highly dependent on the number of livestock. By 2023, the beef cattle breeds in the Republic of Kazakhstan will comprise up to 70% domestic breeds, specifically Kazakh white-headed (60%) and Auliekolskaya (10%), while the remaining 30% will consist of imported breeds, including Aberdeen Angus (18%), Hereford (8%), and Kalmyk (2%) [32,33]. The Republican Chambers of the meat sector have recorded about 1195 livestock enterprises with various ownership forms that are involved in livestock breeding, both domestic and imported, as depicted in Figure 1.

After analyzing the data in Figure 1, it can be observed that the leading chambers include the following:

- For Kazakh white-headed cattle, 804 enterprises (67.2%) are registered, with Kostanay accounting for 1.4% (12 enterprises) and North Kazakhstan accounting for 3.7% (30 enterprises).
- For Hereford cattle, 130 enterprises (10.8%) are registered, with Kostanay accounting for 6.9% (9 enterprises) and North Kazakhstan accounting for 7.6% (10 enterprises).
- For Auliekolskaya cattle, 110 enterprises (9.2%) are registered, with Kostanay accounting for 19% (21 enterprises) and North Kazakhstan accounting for 7.2% (8 enterprises).
- For Aberdeen Angus cattle, 106 enterprises (8.8%) are registered, with Kostanay accounting for 7.5% (8 enterprises) and North Kazakhstan accounting for 15% (16 enterprises).

In contrast to the leading Chambers, the smallest number of enterprises is registered for the Kalmyk cattle breed, accounting for 3.7% (45 enterprises), with North Kazakhstan accounting for 4.4% (2 enterprises) in the studied areas.

**Figure 2:**
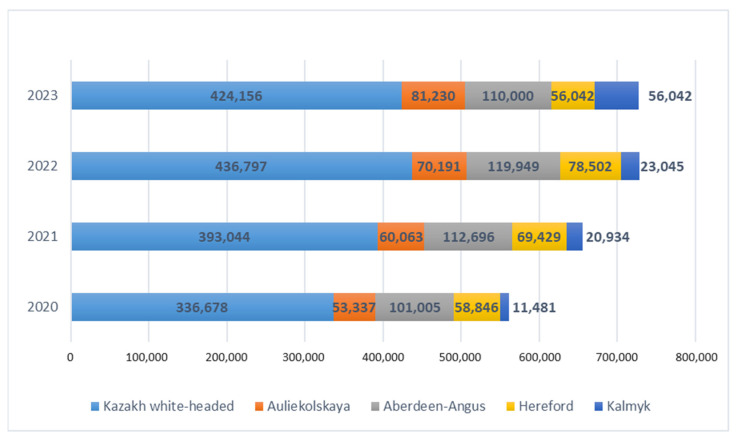
Changes in the numbers of breeding cattle for the studied breeds across the Republic of Kazakhstan from 2020 to 2023, measured in heads.

The monitoring of the number of beef cattle in the Republic of Kazakhstan has shown an increase, reaching 1,095,874 heads in total, including 71.8% or 787,608 heads of the studied breeds, such as the Kazakh white-headed, Hereford, Auliekolsky, Aberdeen Angus, and Kalmyk breeds. Based on the data presented in Table 2 and Figure 2, it can be inferred that the number of cattle is projected to increase by 7.6% in 2023 compared to 2022, 16.7% compared to 2021, and 28.8% compared to 2020 in the Republic of Kazakhstan.

By analyzing the data on the number of cattle separately for the studied breeds from 2020 to 2023, we can conclude that there is a positive trend in respect of the increase in the breeding stock for several breeds. The Kazakh white-headed breed shows an increase of +20.7%; Auliekolskaya shows an increase of 34.3%; Aberdeen Angus shows an increase of 8.2%; Hereford shows an increase of +50.4%; and Kalmyk shows an increase of +20.4%.

### 3.2. Evaluation of Breeding Bulls Based on the Quality of Their Offspring via the Productivity of Cow Daughters Is Important in Determining the Best Genotypes for Breeding

In any breed, animal lines play an important role. These lines typically include exceptional male animals, and over several generations, their best qualities are maintained and improved [34,35,36,37,38]. Working with lines allows for the rapid characterization of the productivity direction of modern breeding stock. Through the selection of cows and appointments for the next calving, while taking the linear affiliation into account, certain populations with distinctive features are formed within the breed, and thus with different genotypes [39,40,41,42,43].

An assessment of the productive characteristics of daughter cows is the main element in determining the best breeding bulls, which is crucial in the breeding of basic farms. The evaluation of live weight, exterior and constitutional features, and milk content is important in animal breeding and contributes to the effective selection and creation of highly productive animals for the future. These estimates are presented in Table 3.

According to the data in Table 3, the evaluation of breeding bulls via the quality of offspring through the productivity of cow daughters shows that superiority in relation to the breed standard is observed in all the studied groups. However, this should be allocated to the group of improvers of proven bulls of Estonian breeding from the farm of Kolos-firma LLP: EE 14465116 ABAJA PORTOS 95283. For these offspring, cow daughters exceeded their peers in live weight, both from their base farm and compared to the peers of the Canadian breeding of the second farm under study (Vishnevskoe LLP). The live weight was the highest at 518 kg, which on average exceeded the breed standard by 19% and the average herd standard of 498.9 kg by 3.7%. The same group should include the offspring of the cow daughters of the Canadian breeding bull (Vishnevskoe LLP: BH BRUIN 54X 1644270), which had higher results in relation to the breed standard by 18.6% and in relation to their herd (500 kg) by 3.1%.

In terms of importance, the second step should include the sires of the Estonian breeding bulls EBA Eagle Bando 1114 KZP156547172, which exceeds the norm of the breed standard by 16.9%; EE 16966079 ABAJA PATRICK 95305, which exceeds the norm by 16.6%; and Kazakh breeding KZP156547191, which is superior by 16.8%.

Another important attribute considered in the breeding of meat cows is the “milk content”, which is estimated via the weight of young animals at the age of 6 months. The results are shown in Table 3.

According to the highest milk-producing ability of cows, it is necessary to distinguish the improvers of sires. These are proven bulls of Estonian breeding (EE 14465116 ABAJA PORTOS 95283), which exceed the breed standard by 23.3% and the herd standard (180.6 kg) by 4.4%. In cows of the bull DK 1989501341 FREDERIK 95220, the excess in relation to the breed standard milk-producing ability was 22.2%; for peers from their own farm, the excess was 9%, and for peers from the farm of Vishnevskoe LLP, the excess was 6.1%.

The same group should include the offspring of a Canadian breeding bull from Vishnevskoe LLP (BH BRUIN 54X 1644270), for which daughters had a milk-producing ability higher than their peers by 5.4%.

### 3.3. Evaluation

For a more objective assessment of the consolidation of desirable hereditary traits (namely, live weight, development of exterior and constitutional features of cows of the studied farms in the context of different genotypes, and characterization of the meat types), the selection and genetic parameters were calculated according to the data in Table 4 and Table 5. This formed the process of evaluating cows depending on different genotypes, including correlation relationships between the traits taken into account, the heritability, the selection differential, and the selection effect.

Upon analyzing the data presented in Table 4, it is notable that all examined groups of cows have yielded correlation trait values that are both positive and negative, within the range *r* = +0.200–+0.600, for the studied productive traits. This observation implies that the breeding of the studied populations based on several crucial productive traits, including “live weight”, “exterior-constitutional features”, and “milk-producing ability “, is judicious.

However, the findings in Table 4 also indicate a negative correlation among the values of productive traits such as “live weight × assessment for the exterior”, “live weight × milk content”, and “estimates for the exterior × milk content” (i.e., −0.200–−0.400), observed in cows of Estonian, Canadian, and Kazakh breeding for individual proven bulls. Nonetheless, this negative correlation is tenable in the case of purebred breeds of farm animals and has a positive impact.

Upon analyzing the data presented in Table 5, it is noteworthy that the studied groups of cows with different genotypes exhibited the distribution of the relationships “live weight × measurements” and “live weight × score for the overall assessment of the exterior”, based on the characteristics of the degrees of the heritability coefficient for the studied productive traits [43,44].

Tracing the obtained values in accordance with Table 5, based on the coefficient of heritability in cows, depending on their origin, one can identify the improver, neutral, useful, and degrader traits of breeding bulls. These findings can be taken into account in the selection of breeds or rejected, potentially contributing to the enhancement of breeding practices.

It is notable that a high degree of heritability coefficient (*h*^2^ = 0.60–1.0, *p* < 0.95) was observed in both farms for the productive characteristics of cows, such as live weight and milk content in bulls of Estonian breeding, including DK 1989501341 FREDERIK 95220 and EE 16966079 ABAJA PATRICK 95305; Canadian breeding JL DISTRICT 0311 1594050; and Kazakh breeding KZP156547191 and Argus KZT155778964.

It can be observed that the average degree of inheritance (*h*^2^ = 0.25–0.59, *p* < 0.95) concerning the productive characteristics of cows, such as live weight and milk content, can be traced in bulls of Estonian breeding, including DK 1989501341 FREDERIK 95220 and EBA Eagle Bando 1114 KZP156547172; Canadian breeding BH BRUIN 54X 1644270 and S7R BARRISTER 45X 1639080; and Kazakh breeding KZP156547191 and Henri KZT157789649.

A low inheritance degree (*h*^2^ = 0.05–0.25, *p* < 0.995) is observed in a bull of Estonian breeding from Kolos-firma LLP, i.e., EE 14465116 ABAJA PORTOS 95283.

Based on the obtained values of the heritability coefficient within “*h*^2^ > 0.3 > 0.7”, it can be concluded that breeding practices in Kolos-firma LLP and Vishnevskoye LLP are both fixed and effective. The likelihood of predicting offspring with the best genotypes from phenotypically superior parents ranges from 30 to 60%, indicating a promising approach to breeding in the studied populations.

To determine the annual forecast of an increase in productive traits in cows, it is imperative to determine the value of the selection effect. However, prior to this, the selection differential needs to be calculated, which refers to the deviation of the trait indicator’s value in the selected (experimental) group from its average value in the herd. Based on the data presented in Table 4, it can be inferred that the breeding differential (*Sd*) in all bull sires ranged from 33.5 to 42.3 in live weight, falling within the normal range. Nonetheless, superiority can be traced in the bull of Estonian breeding (EBA Eagle Bando 1114 KZP156547172) based on “live weight” and in the bull of Canadian breeding (BH BRUIN 54X 1644270) based on “milk-producing ability “.

According to the study, the inheritance of individual characteristics in the majority of bull sires has a positive correlation between the considered productive traits in daughters of cows of Estonian breeding. The study shows that this correlation is 78.6% in the farm of Kolos-firma LLP. The bulls that should be distinguished are EE 14465116 ABAJA PORTOS 95283, DK 1989501341 FREDERIK 95220, and EE 16966079 ABAJA PATRICK 95305. The sire bulls of Canadian breeding show a correlation of 74.5% (Vishnevskoe LLP): BH BRUIN 54X 1644270 and JL DISTRICT 0311 1594050. Similarly, daughter cows from bulls of Kazakh breeding show a correlation of 21.4%, i.e., Kolos-firma LLP: KZP156547191; in Vishnevskoe LLP, this correlation is 25.5% for Henri KZT157789649 and Argus KZT155778964.

Based on the study conducted, it was found that the inheritance of individual characteristics in most bull sires has a positive correlation with the productive traits of the daughters of cows in Estonian breeding. Specifically, in the farm of Kolos-firm LLP, this correlation was 78.6%. Bulls that should be distinguished include EE 14465116 ABAJA PORTOS 95283, DK 1989501341 FREDERIK 95220, and EE 16966079 ABAJA PATRICK 95305. In addition, sire bulls of Canadian breeding show a correlation of 74.5% (Vishnevskoe LLP), wherein BH BRUIN 54X 1644270 and JL DISTRICT 0311 1594050 are noteworthy. Similarly, daughter cows from bulls of Kazakh breeding show a correlation of 21.4% (Kolos-firma LLP, KZP156547191); in Vishnevskoe LLP, Henri KZT157789649 and Argus KZT155778964 exhibit a correlation of 25.5%.

The lowest value was obtained for a bull of the Estonian selection of Kolos-firma LLP, namely EE 14465116 ABAJA PORTOS 95283, which may be due to the low variability of the studied productive traits in cows.

In conclusion, it is important to have knowledge of the accompanying breeding and genetic parameters that determine the future productivity of the breeding stock, including live weight, exterior and constitutional features, and milk-producing ability. This allows for better control of the use of valuable proven bulls that affect the future high productivity of their offspring and the results of targeted selection. This, in turn, will result in both breeding and economic efficiency.

## 4. Discussion

Recently, for the production of high-quality beef, Kazakhstan began to import Aberdeen Angus cattle from Estonia, Canada, and other countries [10,28,29,30]. In this regard, there was a necessity to comparatively estimate the cows’ productive qualities depending on different genotypes for the purpose of cow selection and revealing successful bull-improvers [16,26].

Nowadays, in order to achieve progress, the evaluation of breeding bulls is of great importance, because in large-scale breeding, it is desirable to accelerate the reproduction process and create animals that have high productive values and are well adapted to innovative technologies [21,22,23,24]. Various evaluation techniques are used to select animals that will participate in further herd reproduction.

Our results agree with similar studies of leading authors, who also state that the most effective selection method is the evaluation of proven bulls through comparison of their daughter cows with their peers. This provides corrections for the number of accounted daughters and peers, which increases the reliability of the prediction of the breeding value of both cows and their sires/bull-improvers [45,46,47].

In order to improve the profitability and quality of beef production in the northern region of Kazakhstan, it is recommended that the genetic potential of the Aberdeen Angus breed is optimized. This can be achieved by selecting highly productive breeding stock with a pronounced meat type that consistently transmit these desirable traits from their ancestors on the paternal side. The use of such animals can significantly influence the qualitative transformation of the herd, leading to increased profitability and improved meat quality. Additionally, the evaluation of bulls based on the quality of offspring through daughters is of utmost importance in the successful implementation of artificial insemination and the widespread use of deep-frozen seed in Kazakh meat-breeding work.

Thus, the analysis of the studied method of evaluation of proven bulls, and of the quality of offspring daughter cows compared with their peers in relation to productive qualities, has shown that productive traits should be taken into account as criteria for the evaluation of mother cows.

The continuous improvement and introduction of such evaluation systems in beef cattle breeding at the state level are prerequisites for progressive breeding work (increasing the potential of breeding and productive qualities of cattle) and, as a consequence, the profitability of the industry.

To increase profitability and improve the quality of beef in northern Kazakhstan, we recommend using the genetic potential of Aberdeen Angus stock of exotic origin more effectively.

## 5. Conclusions

The determination of the pedigree value of bulls of the Aberdeen Angus breed in the northern region of Kazakhstan on the basis of productive characteristics of their daughter cows is an opportunity to provide better control over valuable bull-improvers of leading paternal lines of exotic selection and over their use in the improvement of herd reproduction in local Kazakhstan breeding for the future.

## Figures and Tables

**Table 1:** Distribution of cows depending on origin.

Group of Cows	Proven Bull	n
Kolos-firma LLP		
Estonian breed		
I	DK 1989501341 FREDERIK 95220	11
	EBA Eagle Bando 1114 KZP156547172	17
	EE 14465116 ABAJA PORTOS 95283	10
	EE 16966079 ABAJA PATRICK 95305	10
Kazakhstani breed		
II	KZP156547191	17
Vishnevskoe LLP		
Canadian breed		
III	BH BRUIN 54X 1644270	10
	S7R BARRISTER 45X 1639080	13
	JL DISTRICT 0311 1594050	13
Kazakhstani breed		
IV	Henry KZT157789649	10
	Argus KZT155778964	10

**Table 2:** The number of breeding cattle of the studied breeds in the Republic of Kazakhstan for 2020–2023, heads.

Breed	Year				2023 Compared to 2020 in %
	2020	2021	2022	2023	
Kazakh white-headed	336,678	393,044	436,797	424,156	+20.7
Auliekolskaya	53,377	60,063	70,191	81,230	+34.3
Aberdeen Angus	101,005	112,696	119,949	110,000	+8.2
Hereford	58,846	69,429	78,502	116,180	+50.4
Kalmyk	11,481	20,934	23,045	56,042	+20.4
Total:	561,387	656,166	728,484	787,608	+28.8

**Table 3:** Evaluation of breeding bulls based on the quality of offspring through the productivity of cow daughters (*X ± M_X_*).

	Breeding Bull	n	Live Weight, kg	Assessment of the Constitution and Exterior Is Measured in Points	Milk-Producing Ability, kg
	Kolos-firma LLP				
	Estonian breed				
I	DK 1989501341 FREDERIK 95220	11	482.8 ± 2.4	28.9 ± 0.7	186.3 ± 2.5
	EBA Eagle Bando 1114 KZP156547172	17	505.2 ± 2.4	29.9 ± 0.6	182.0 ± 1.5
	EE 14465116 ABAJA PORTOS 95283	10	518.0 ± 3.1	28.6 ± 2.8	188.9 ± 0.7
	EE 16966079 ABAJA PATRICK 95305	10	503.4 ± 3.6	28.8 ± 1.4	178.8 ± 1.4
Kazakhstani breed					
II	KZP156547191	17	504.5 ± 3.8	29.8 ± 3.2	175.6 ± 2.6
Vishnevskoe LLP					
Canadian breed					
III	BH BRUIN 54X 1644270	10	515.9 ± 2.4	28.6 ± 0.7	187.2 ± 0.4
	S7R BARRISTER 45X 1639080	13	498.2 ± 3.8	28.8 ± 2.8	178.5 ± 1.6
	JL DISTRICT 0311 1594050	13	505.2 ± 2.6	29.8 ± 2.3	179.5 ± 0.4
Kazakhstani breed					
IV	Henri KZT157789649	10	494.0 ± 2.6	29.5 ± 1.2	175.3 ± 1.1
	Argus KZT155778964	10	503.4 ± 5.1	28.6 ± 2.7	175.6 ± 3.8
	Breed standard		420	30	145

n—number of animals. *X*—is the weighted average. *M_X_*—reliability of the weighted average.

**Table 4:** The correlation coefficients (*r*) of productive traits of cows depending on different origins.

Breeding Bull	Features			Reliability, (*p*)
	Live Weight, kg × Exterior Score, Point	Live Weight, kg × Milk-Producing Ability, kg	Assessment for the Exterior, Point × Milk-Producing Ability, kg	
Kolos-firma LLP				
DK 1989501341 FREDERIK 95220	+0.420	+0.400	+0.550	*p* < 0.95
EBA Eagle Bando 1114 KZP156547172	+0.600	+0.500	+0.300	*p* < 0.95
EE 14465116 ABAJA PORTOS 95283	+0.300	+0.550	+0.300	*p* < 0.95
EE 16966079 ABAJA PATRICK 95305	+0.300	−0.300	−0.200	*p* < 0.95
KZP156547191	+0.300	−0.400	−0.300	*p* < 0.95
Vishnevskoe LLP				
BH BRUIN 54X 1644270	+0.230	−0.300	+0.420	*p* < 0.95
S7R BARRISTER 45X 1639080	+0.520	+0.0450	+0.500	*p* < 0.95
JL DISTRICT 0311 1594050	+0.450	+0.500	−0.400	*p* < 0.95
Henri KZT157789649	+0.500	+400	+500	*p* < 0.95
Argus KZT155778964	−0.200	−200	+500	*p* < 0.95

*r*—correlation coefficient. *P*—the reliability of the difference in average values.

**Table 5:** Forecasting the improvement of productive traits based on the results of the evaluation of cows, depending on origin.

Indicator	Variety of Breeding Bulls									
	Kolos-Firma LLP					Vishnevskoe LLP				
	DK 1989501341 FREDERIK 95220	EBA Eagle Bando 1114 KZP156547172	EE 14465116 ABAJA PORTOS 95283	EE 16966079 ABAJA PATRICK 95305	KZP156547191	BH BRUIN 54X 1644270	S7R BARRISTER 45X 1639080	JL DISTRICT 0311 1594050	Aнpи KZT157789649	Apгyc KZT155778964
Live weight, kg										
*h* ^2^	0.4	0.4	0.2	0.6	0.5	0.4	0.3	0.5	0.5	0.6
*Sd*	35.4	42.3	36.5	32.8	40.5	35.8	33.5	40.3	37.6	39.4
*SE*	30.6	28.5	24.6	32.5	31.4	28.5	34.2	30.5	32	29.5
Milk-producing ability, kg										
*h* ^2^	0.6	0.5	0.4	0.3	0.6	0.4	0.3	0.6	0.4	0.6
*Sd*	10.6	8.5	8.2	9.4	9.6	12.3	9.4	10.4	8.1	9.2
*SE*	12.5	10.5	7.3	8.7	8.8	12.8	8.5	8.6	10.4	9.7

*h*^2^—heritability coefficient. *Sd*—selection differential. *SE*—selection effect.

## Data Availability

All relevant data are presented within the paper.
